# First principles calculation of the nonhydrostatic effects on structure and Raman frequency of 3C-SiC

**DOI:** 10.1038/s41598-018-29666-2

**Published:** 2018-07-26

**Authors:** Liu Lei, Yi Li, Liu Hong, Li Ying, Zhuang Chun-Qiang, Yang Long-Xing, Liu Gui-Ping

**Affiliations:** 1 Key Laboratory of Earthquake Prediction, Institute of Earthquake forecasting, China Earthquake Administration, Beijing, 100036 China; 2 National Key Laboratory of Shock Wave and Detonation Physics, Mianyang, 621900 China; 3 Institute of Microstructure and Properties of Advanced Materials, Beijing University of Technology, Beijing, 100124 China

## Abstract

For understanding the quantitative effect of nonhydrostatic stress on properties of material, the crystal structure and Raman spectra of 3C-SiC under hydrostatic and nonhydrostatic stress were calculated using a first-principles method. The results show that the lattice constants (*a*, *b*, and *c*) under nonhydrostatic stresses deviate those under hydrostatic stress. The differences of the lattice constants under hydrostatic stress from nonhydrostatic stresses with differential stress were fitted by linear equation. Nonhydrostatic stress has no effect on density of 3C-SiC at high pressure, namely the equations of state of 3C-SiC under hydrostatic stress are same as those under nonhydrostatic stress. The frequencies and pressure dependences of LO and TO modes of 3C-SiC Raman spectra under nonhydrostatic stress are just same as those under hydrostatic stress. Under nonhydrostatic stress, there are four new lines with 361, 620, 740, and 803 cm^−1^ appeared in the Raman spectra except for the LO and TO lines because of the reduction of structure symmetry. However the frequencies and pressure dependences of the four Raman modes remain unchanged under different nonhydrostatic stresses. Appearance of new Raman modes under nonhydrostatic stress and the linear relationship of the differences of lattice constants under hydrostatic and nonhydrostatic stresses with differential stress can be used to indicate state of stress in high pressure experiments. The effect of nonhydrostatic stress on materials under high pressure is complicated and our calculation would help to understanding state of stress at high pressure experiments.

## Introduction

Diamond anvil cells (DACs) have long been extensively used to generate high pressure on materials for research purposes. Recently, extreme pressures to 750 GPa has been achieved using DACs with improved design^1^. However, maintaining hydrostatic conditions under such extreme pressures has long been a great challenge. Generally at low pressures, hydrostatic conditions in DACs can be well maintained by proper experimental set-ups, such as gaskets thickness, sample size, sample chamber diameter, and choice of pressure-transmitting media (PTM). But at extreme high pressure, pressure induced solidification of the PTM is inevitable and effects of non-hydrostatic stress caused by PTM solidification will become more and more ineligible with increasing pressure. The stress in the PTM and the sample will both begin to depart from hydrostatic under extreme pressures.

Nonhydrostatic stress has been reported to have unique influence on material. Powder diffraction lines tend to broaden significantly under nonhydrostatic stresses^2,3^. The phase transformations of a number of compounds show strong dependence of transformation pressure and sequence on nonhydrostatic stress^4–12^. The pressure dependencies of crystal parameters of aegirine (NaFeSi_2_O_6_) measured by the diffraction experiments showed anomalies at 12.62 GPa and above under nonhydrostatic stress^13^. The decrease of magnetic moment of bcc iron with increasing pressure under nonhydrostatic stress is faster than those under hydrostatic stress^14^. The density, lattice strain and elastic constants of forsterite under differential stress are different from those of under hydrostatic stress and the difference increase with increasing differential stress^15^. The Raman modes shifting their frequencies to either higher or lower values, relative to its equivalent value under hydrostatic pressure, depend on the state of differential stress^16^.

Studies of the state of nonhydrostatic stress in high pressure experiment have thus received considerable attentions. Two distinct theories have been proposed with the anisotropic elasticity theory (AET)^17–22^ and the isotropic elasticity theory (IET)^23–28^. These theories were used to analyze the state of nonhydrostatic stress in high pressure experiments by deriving relationship of lattice strain, d-spacing, and the diffraction data. Estimation of nonhydrostatic stress using IET and AET requires the knowledge of the crystal structure parameters and elastic moduli of the material under hydrostatic stress. Due to the difference of the lattice strain and elastic constant of materials under non-hydrostatic and hydrostatic stress^15,16^, quantitatively studies of lattice parameters under nonhydrostatic stress are essential understanding the state of nonhydrostatic stress under high pressure. However, quantitatively investigation is both time consuming and difficult to practice due to the poor repeatability of high-pressure experiments. First-principles methods made great success in calculating material properties^29–37^ including the state of nonhydrostatic (differential) stress and its effects at atomic scale^15,16,38–45^. Therefor it is suitable used for this works.

The cubic 3C-SiC with the zinc blende crystal possesses the nearest to the diamond structure in both carbon and silicon. The 3C-SiC possesses high chemical stability and high bulk and shear modulus, 220 GPa and 183 GPa, respectively^46–50^. The 3C-SiC crystal transforms to a rocksalt phase at 66GPa^46^–100GPa^51^ and maintains stable till 2373 K^52^. Meanwhile, as a cubic crystal, it is easy to clarify the relationship between lattice strain and nonhydrostatic stress. So 3C-SiC is an ideal sample for studying the effect of differential stresses on materials properties. Here we report our first-principle calculation about the effect of nonhydroustatic stress on crystal structure and Raman spectrum of 3C-SiC.

## Results and Discussion

### Lattice parameters

**Figure 1:**
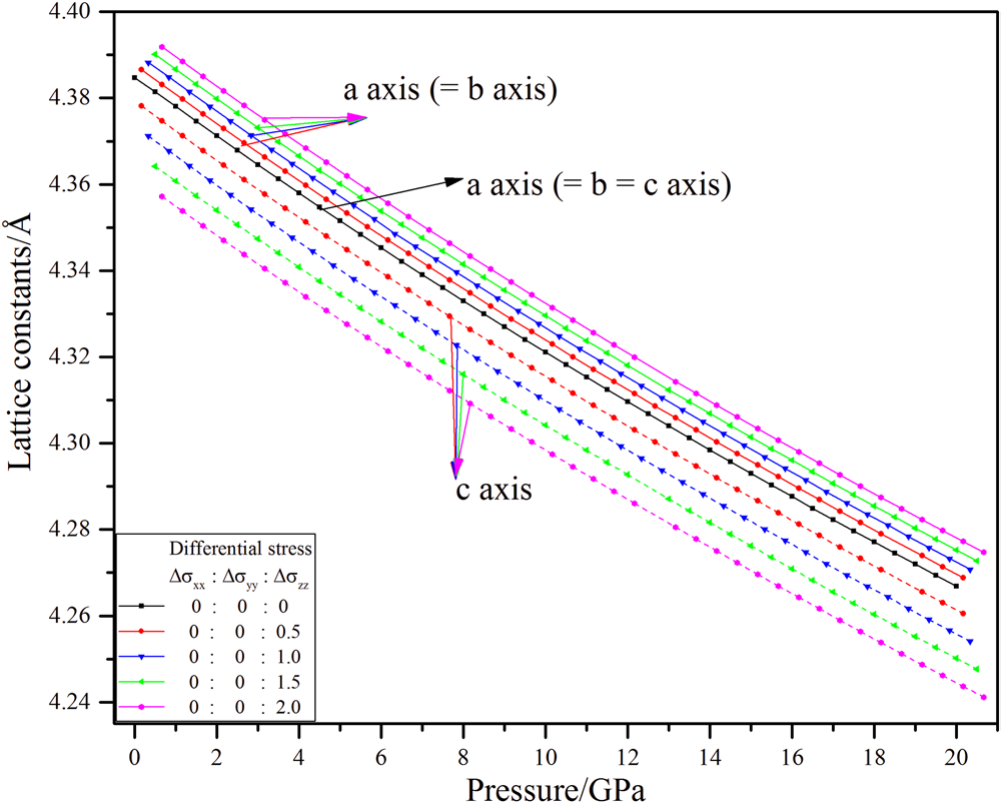
Lattice constants of 3C-SiC under different stresses.

Figure 1 shows lattice constants of 3C-SiC as a function of stress. Additional pressures were put following ***z*** direction (***c***-axis) for producing the nonhydrostatic stresses during pressuring. As a cubic symmetrical crystal, the lattice constants a, b, and c are equal and all three bond angles (α, β, and γ) are 90°. However under nonhydrostatic stresses, the lattice constants of 3C-SiC are not equal (a = b ≠ c), so the 3C-SiC cannot keep cubic symmetry under nonhydrostatic stress. The lattice constants under nonhydrostatic stresses deviate those under hydrostatic stress. The ***a*** and ***b*** (***a*** = ***b***) are larger than their equivalent hydrostatic values under nonhydrostatic stresses; however, those of ***c*** are smaller. Namely, the effect of nonhydrostatic stresses on the lattice constants shows the Poisson effect that consistent with previous results^15,16^.

**Figure 2:**
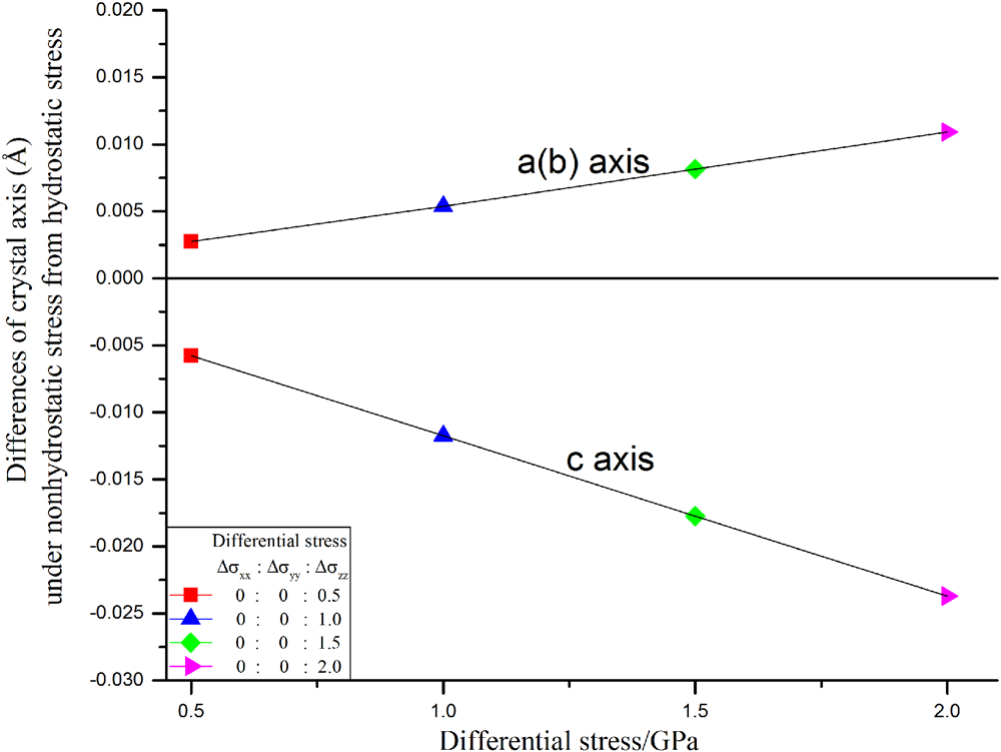
The differences of lattice constants under hydrostatic and nonhydrostatic stresses.

The differences of lattice constants of 3C-SiC between nonhydrostatic and hydrostatic stresses were presented in Fig. 2. Obviously, effect of nonhydrostatic stress on lattice constants linearly increases with increasing differential stress. The difference of the lattice constants (***a***, ***b***, and ***c***) under hydrostatic stress from nonhydrostatic stresses with differential stress were fitted by a linear equation as follows:

1 $${\rm{For}}\,{\boldsymbol{a}}\,{\rm{and}}\,{\boldsymbol{b}},\,{\bf{D}}=0.0054{{\bf{S}}}_{{\bf{D}}}-1{\rm{E}} \mbox{-} 5,\,{{\rm{R}}}^{2}=1$$

2 $${\rm{For}}\,{\boldsymbol{c}},\,{\bf{D}}=0.012{{\bf{S}}}_{{\bf{D}}}-2{\rm{E}} \mbox{-} 4,{{\rm{R}}}^{2}=1$$

where **D** (unit, Å) indicates the differences in lattice constants under hydrostatic and nonhydrostatic stresses; **S**_**D**_ (unit, GPa) indicates the amount of differentials stress.

The effect of nonhydrostatic stress on ***c*** axis is about 2.2 times that of ***a*** and ***b*** axis because ***a*** and ***b*** axis simultaneously increase, however only ***c*** axis decrease under nonhydrostatic stresses. Those linear relations are help to understand state of nonhydrostatic stress in high pressure experiments.

### Density/Equation of State

**Figure 3:**
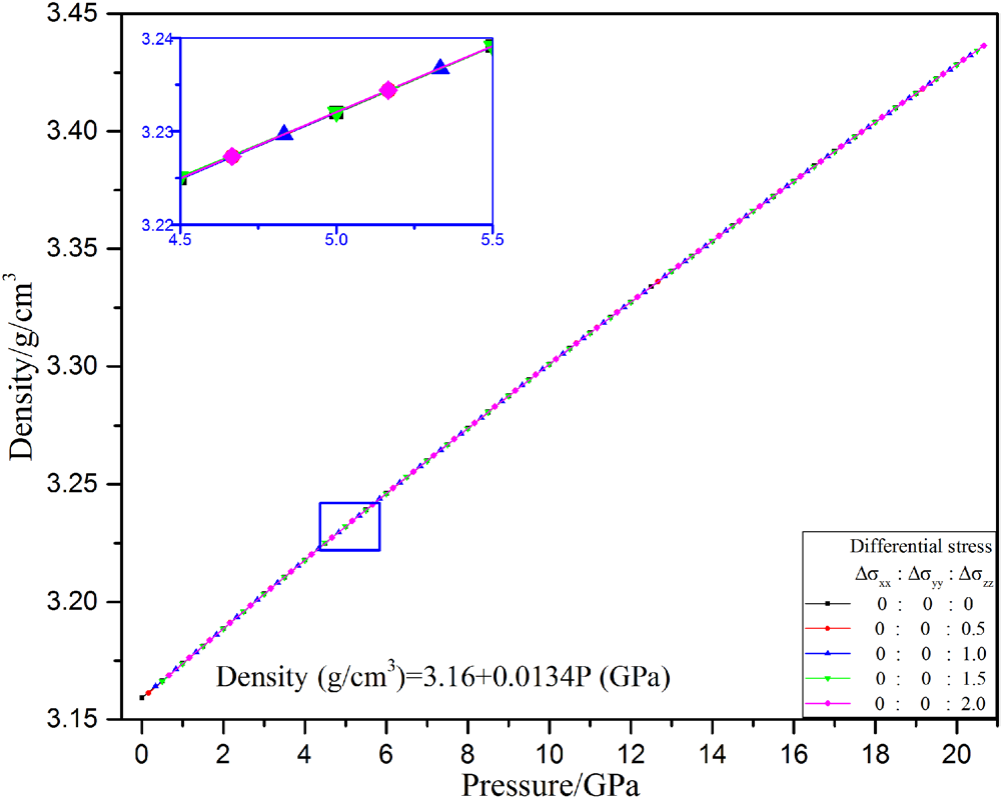
Variation of density of 3C-SiC with different stress conditions.

The densities of 3C-SiC under hydrostatic and nonhydrostatic stresses are shown in Fig. 3. It is very hard to find some differences among the densities under hydrostatic and nonhydrostatic stresses. Even from the partial enlarged figure, the densities under different stress also are identical. They all linearly increase with increasing pressure. The linear equation was used to describe the relationship of density and pressure as

3 $${\boldsymbol{Density}}\,({\boldsymbol{g}}/{\boldsymbol{c}}{{\boldsymbol{m}}}^{{\rm{3}}})={\boldsymbol{3.16}}+{\boldsymbol{0}}{\boldsymbol{.0134P}}\,({\boldsymbol{GPa}})$$

The result indicates that nonhydrostatic stress has no effect on cell volume of 3C-SiC at high pressure, namely the equations of state of 3C-SiC under hydrostatic stress are same as those under nonhydrostatic stress. The result is different with the effect of nonhydrostatic stress on density of forsterite^15^ and calcite^16^. The density of calcite and forsterite increases or decreases under different differential stress, relative to its equivalent values under hydrostatic pressure, depend on the state of differential stress. So the effect of nonhydrostatic stress on density of materials under high pressure is complicated. More works need to be done on effect of nonhydrostatic stress on equation of state of materials.

### Raman modes

Raman scattering conveys structural information about the lattice vibrational properties of solids. Because of difference in the electronegativity of Si and C, the optical modes of 3C-SiC at Γ point of Brillouin zone are split into two degenerate transverse optical modes (TO) and a nondegenerate longitudinal optical mode (LO)^53^. The pressure derivative of the LO and TO modes and LO-TO splitting that got in this work and previous researches under hydrostatic stress are listed in Table 1. The Raman frequencies of 3C-SiC were obtained by experiment^53–55^ with 795.9–797.7 cm^−1^ (TO) and 972.9–973.6 cm^−1^ (LO) and by calculation^55,56^ with 783–784 cm^−1^ (TO) and 956–958 cm^−1^ (LO). Our calculated TO and LO modes agree with the previous experimental and calculated data and the differences are within 3%. The TO and LO lines shift to higher frequencies with increasing pressure. The LO-TO splitting also increase with increasing pressure because of an unusually increase of the ionicity of 3C-SiC under pressure that caused by charge transfer from Si to C^57^.

**Table 1:** Pressure dependence of Raman frequencies of 3C-SiC under hydrostatic stress. Phonon frequencies ν_0_ are in cm^−1^ and dν/dP in cm^−1^/GPa.

	ν_0_	dν/dP	ν_0_	dν/dP	ν_0_	dν/dP	ν_0_	dν/dP
TO	797.7	3.88	797.2	3.46	774.1	3.65	783.7	3.813
LO	973.6	4.59	973.8	4.27	942.3	4.25	958.5	4.417
LO-TO	175.9	0.654	176.6	0.812	168.6	0.578	174.8	0.604
Method	Expt.				Calc.			
References	^55^		^53^		This works		^55^	

Note: The results refer to fits of linear functions for experimental data.

**Figure 4:**
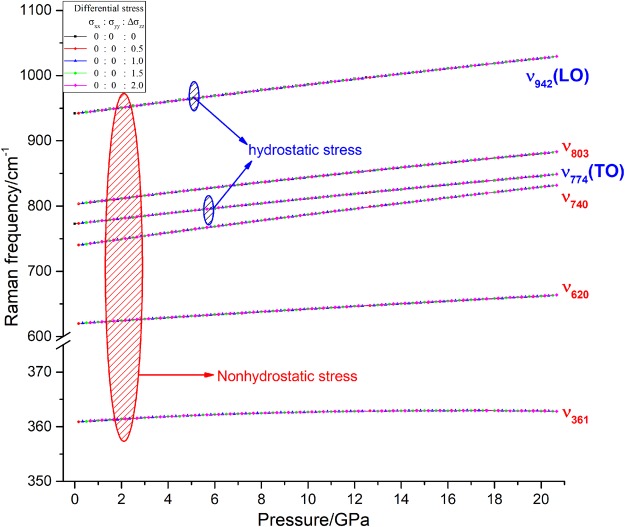
Variation of Raman frequencies of 3C-SiC with pressure.

The pressure dependences of the Raman frequencies of 3C-SiC under hydrostatic and nonhydrostatic stress are displayed in Fig. 4. Under hydrostatic stress, only two Raman frequencies of 3C-SiC show in the Raman spectrum, namely LO and TO lines. However under nonhydrostatic stress, there are four new lines with 361, 620, 740, and 803 cm^−1^ appeared in the Raman spectra except for the LO and TO lines because of the reduction of crystal structure symmetry. The pressure dependences of LO and TO modes under nonhydrostatic stress are just same as those under hydrostatic stress. For the 4 new Raman modes (361, 620, 740, and 803 cm^−1^), their frequencies linear increase with increasing pressure and their pressure derivative are 0.1, 2.1, 4.6, and 3.9 cm^−1^/GPa.

**Figure 5:**
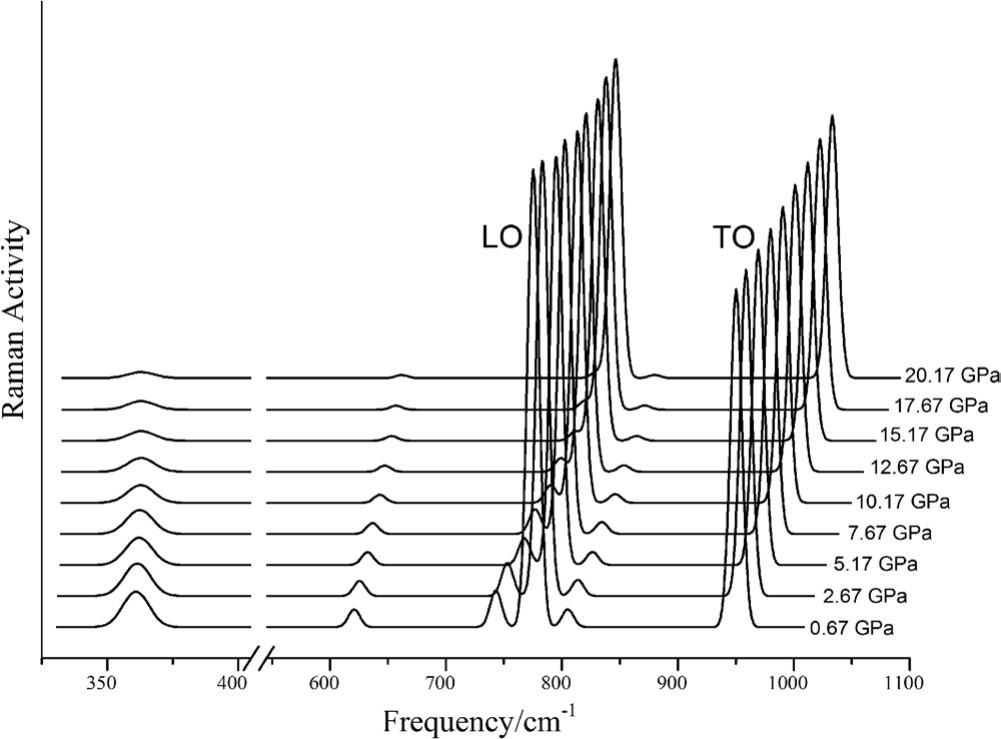
Raman spectrum of 3C-SiC under nonhydrostatic stress.

**Figure 6:**
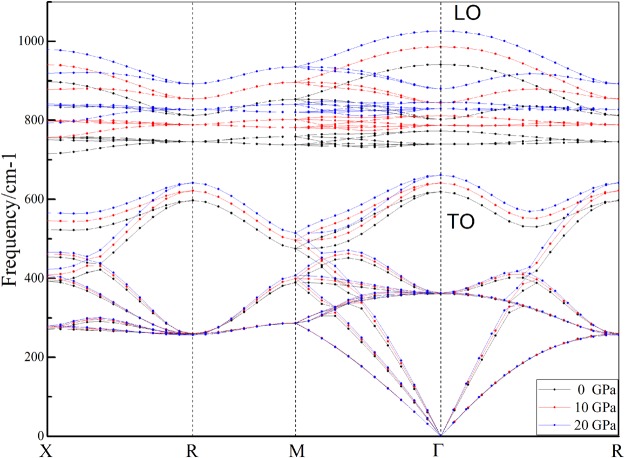
Phonon dispersions of 3C-SiC along the main symmetry directions.

However the frequencies and pressure dependences of the 4 Raman modes remain unchanged under these 4 different nonhydrostatic stress states. Namely for all Raman modes of 3C-SiC, the states of nonhydrostatic stress have no effect on frequency and pressure dependence. However the activity of 4 new Raman frequencies decrease with increasing pressure and mean the effect of nonhydrostatic the Raman spectrum weaken under high pressure (Fig. 5). Though nonhydrostatic stresses have no effect on Raman frequency and its pressure derivative of 3C-SiC, the new appeared Raman modes under nonhydrostatic stress can be used to indicate state of stress in the high experiment. Therefor those results can help to understanding state of stress at high pressure experiments. Because the differential stresses have no effect pressure dependence of the Raman frequencies, we only list the phonon dispersion of 3C-SiC under hydrostatic stress with 0, 10, and 20 GPa as Fig. 6. The phonon dispersion relation spectrum show consistent results with pressure dependences of the Raman frequencies.

## Computational Method

### Computational background

First principles calculations were performed by density functional perturbation theory (DFPT)^58^, density functional theory (DFT)^59,60^ and plane wave pseudopotential technique, as implemented in CASTEP-2017 codes^61^. The generalized gradient approximation (GGA-PBE) was used to describe exchange-correlation interactions^62^. Norm Conserving Pseudopotential^63^ was employed to model electron-ion interaction. A 8 × 8 × 8 Monkhorst Pack grid of k points was adopted for sampling Brillouin zone. A convergence criterion of 5 × 10^−7^a.u. on the total energy was used in the self-consistent field (SCF) calculations. The energy cutoff for plane wave basis was chosen as 900 eV. Geometrically-optimized convergence criterion used in energy, maximum force, maximum stress, and maximum displacement are 5 × 10^−6^ eV/atom, 0.01 eV/Å, 0.02 GPa, and 5 × 10^−4^ Å, respectively.

The structure and Raman spectroscopy of 3C-SiC at given pressures were calculated by optimizing simultaneously both lattice constants and atomic positions based on the consideration that Hellmann–Feynman forces and stresses applied respectively on nuclei and lattice parameters were minimized, respectively^64^. Spatial derivatives of the macroscopic polarization were calculated numerically along eigenvectors of each Raman active phonon mode according to the polarization for each displacement using linear response formalism^65^. Once these derivatives are determined, the Raman cross-section through appropriate averaging space can be calculated. Further details can be found in Porezag and Pederson^66^ and Refson *et al*.^58^.

**Figure 7:**
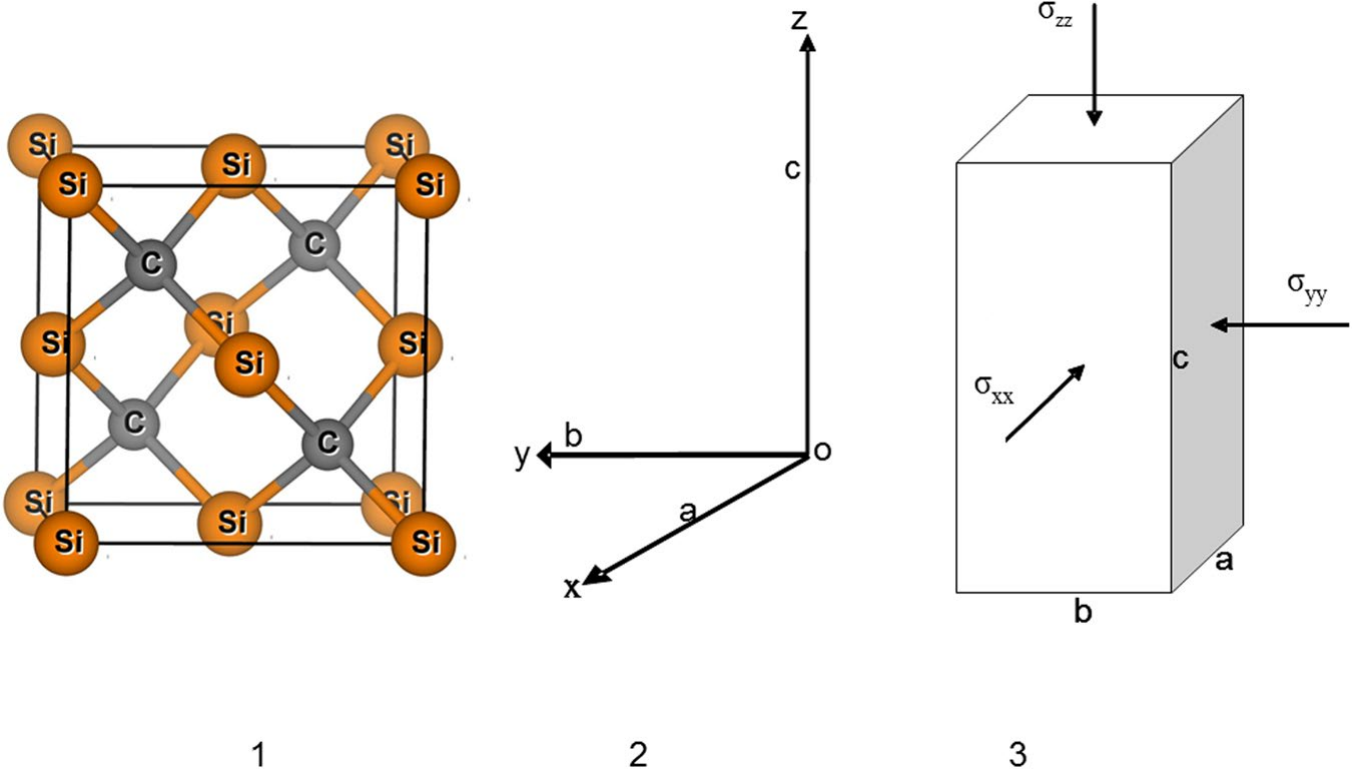
Atomic structure of 3C-SiC and schematic diagram of differential stress project. (1) Atomic structure of 3C-SiC, (2) Lattice constants and directions, and (3) Applied stresses (σ_xx_, σ_yy_, and σ_zz_).

Different stresses were applied to the crystal along ***a***, ***b***, and ***c*** axis directions (i.e., x, y, and z direction). They were marked as σ_xx_, σ_yy_, and σ_zz_, which were schematically illustrated in Fig. 7. Thus, the equivalent hydrostatic pressure (***P***, GPa) applied to crystals is (σ_xx_ + σ_yy_ + σ_zz_)/3. When σ_xx_ = σ_yy_ = σ_zz_, the pressure is hydrostatic; when σ_xx_ ≠ σ_yy_ or σ_zz_, the pressure is nonhydrostatic. Therefore, if σ_xx_ is equal to ***P***_***1***_, σ_yy_ is equal to ***P***_***1***_, and σ_zz_ is equal to ***P***_***1***_ + ***x*** GPa, then ***P*** is equal to ***(3P***_***1***_ + ***x)/3*** GPa, differential stress is ***△***σ_xx_ = σ_xx_ − ***P*** = −***x***/3 GPa, ***△***σ_yy_ = σ_yy_ − ***P*** = −***x***/3 GPa, and ***△***σ_zz_ = σ_zz_ − ***P*** = 2***x***/3 GPa. The largest stress difference among σ_zz_, σ_yy_, and σ_xx_ is ***x*** GPa. Here, effects of five sets of different stresses including hydrostatic pressure (namely, x = 0, 0.5, 1, 1.5 and 2 GPa) on the structure and Raman spectrum of 3C-SiC were calculated.

### Benchmark calculation

To assess the performance of the DFT and DFPT total-energy approach used here, we performed test calculations on the structural and elastic of 3C-SiC (Table 2). The close agreement between previous results and our calculations demonstrates the validity of our computational method. The results indicate that the calculation approach well replicates the properties of 3C-SiC.

**Table 2:** Structural, elastic, and Raman characters of 3C-SiC under ambient condition.

Lattice constant (Å)			
*a*	Method	Reference	
4.361	Calc.	^46^	
4.365	Calc.	^48^	
4.360	Expt.	^50^	
4.326	Expt.	^47^	
4.348	Calc.	This works	
**Elastic modulus (GPa)**			
Bulk modulus (GPa)	Shear modulus (GPa)		
196.2		Calc.	^67^
212		Calc.	^46^
200		Calc.	^48^
211		Calc.	^49^
225.2		Calc.	^68^
224		Expt.	^50^
248		Expt.	^69^
211.4	183.5	Expt.	^70^
208.7	186.4	Calc.	This works
**Raman frequency**			
TO	LO		
795.9	972.9	Expt.	^54^
797.7	973.6	Calc.	^55^
783	956	Calc.	^56^
772.7	941.1	Calc.	This works
